# The insights into the systematic relationship of *Gastrostyla*-affinitive genera, with report on a new saline soil ciliate genus and new species (Protozoa, Ciliophora)

**DOI:** 10.1186/s12862-020-01659-8

**Published:** 2020-07-29

**Authors:** Xiaoteng Lu, Yuanyuan Wang, Saleh A. Al-Farraj, Hamed El-Serehy, Jie Huang, Chen Shao

**Affiliations:** 1 Laboratory of Protozoological Biodiversity and Evolution in Wetland, College of Life Sciences, Shaanxi Normal University, Xi’an, 710119 China; 2 Research Department for Limnology, Mondsee, University of Innsbruck, Mondseestrasse 9, A-5310 Mondsee, Austria; 3 Institute of Evolution & Marine Biodiversity, Ocean University of China, Qingdao, 266003 China; 4 Zoology Department, King Saud University, Riyadh, 11451 Saudi Arabia; 5 Key Laboratory of Aquatic Biodiversity and Conservation of Chinese Academy of Sciences, Institute of Hydrobiology, Chinese Academy of Sciences, Wuhan, 430072 China

**Keywords:** Convergent evolution, *Gastrostyla*, morphogenesis, new genus, new species, saline soil habitat, 18S rDNA phylogeny

## Abstract

**Background:**

Hypotrichia are a group with the most complex morphology and morphogenesis within the ciliated protists. The classification of *Gastrostyla*-like species, a taxonomically difficult group of hypotrichs with a common ventral cirral pattern but various dorsal and ontogenetic patterns, is poorly understood. Hence, systematic relationships within this group and with other taxa in the subclass Hypotrichia remain unresolved.

**Results:**

18S rRNA gene sequence of a new *Gastrostyla*-like taxon was obtained. Phylogenetic analyses based on the 18S rRNA gene sequences indicate that this ciliate represents a new genus that is closely related to *Heterourosomoida* and *Kleinstyla* within the oxytrichid clade of the Hypotrichia. However, the position of this cluster remains unresolved. All three genera deviate from the typical oxytrichids by their incomplete (or lack of) dorsal kinety fragmentation during morphogenesis. Morphology and morphogenesis of this newly discovered form, *Heterogastrostyla salina* nov. gen., nov. spec., are described. *Heterogastrostyla* nov. gen., is characterised as follows: more than 18 fronto-ventral-transverse cirri, cirral anlagen V and VI develop pretransverse cirri, and dorsal ciliature in *Urosomoida*-like pattern.

**Conclusions:**

Similar to the CEUU-hypothesis about convergent evolution of urostylids and uroleptids, we speculate that the shared ventral cirral patterns of *Gastrostyla*-like taxa might have resulted from convergent evolution.

## Background

Hypotrichia are a group with the most complex morphology and morphogenesis within the ciliated protists. They are thus increasingly recognized as being of significance to the study of cell biology, genetics and ecology [1–15].

Among hypotrichs, *Gastrostyla*-like species are a group of superficially similar taxa that have at least seven frontoventral cirri (derived from anlagen IV–VI) in a more or less continuous slightly oblique row [16–19]. *Gastrostyla*-like forms include species belonging to the genera *Neogastrostyla* Kaur et al., 2019 [20], *Gastrostyla* Engelmann, 1862 [21], *Kleinstyla* Foissner et al., 2002 [22], *Apogastrostyla* Li et al., 2010 [17], *Hemigastrostyla* Song and Wilbert 1997 [19], *Protogastrostyla* Gong et al., 2007 [23], and *Pseudogastrostyla* Fan et al., 2015 [24].

Despite sharing a common ventral cirral pattern, the systematic position of *Gastrostyla*-like species is still problematic [16, 25–28]. Generally, there are two possibilities: (i) *Gastrostyla*-like species were ancestors of the typical 18 FTV-cirri oxytrichids, that is, the 18 FTV-cirral pattern evolved from a *Gastrostyla*-like pattern by a reduction of the cirri originating from anlagen IV–VI [29, 30]; or (ii) *Gastrostyla*-like species evolved from 18 FTV-cirri oxytrichids independently by increasing the number of cirri originating from anlagen IV-VI [16].

In April 2015, an undescribed *Gastrostyla*-like species was isolated from saline soil within the Longfeng Wetland Nature Reserve, a district of Daqing, northern China. Analyses of its morphology and cell division, as well as the small subunit ribosomal DNA (SSU rDNA) sequence, indicate that it represents a new species and a new genus. Phylogenetic analyses of all available *Gastrostyla*-like species were performed.

## Results

### SSU rDNA sequence and phylogenetic analyses (Fig. 1)

**Fig. 1:**
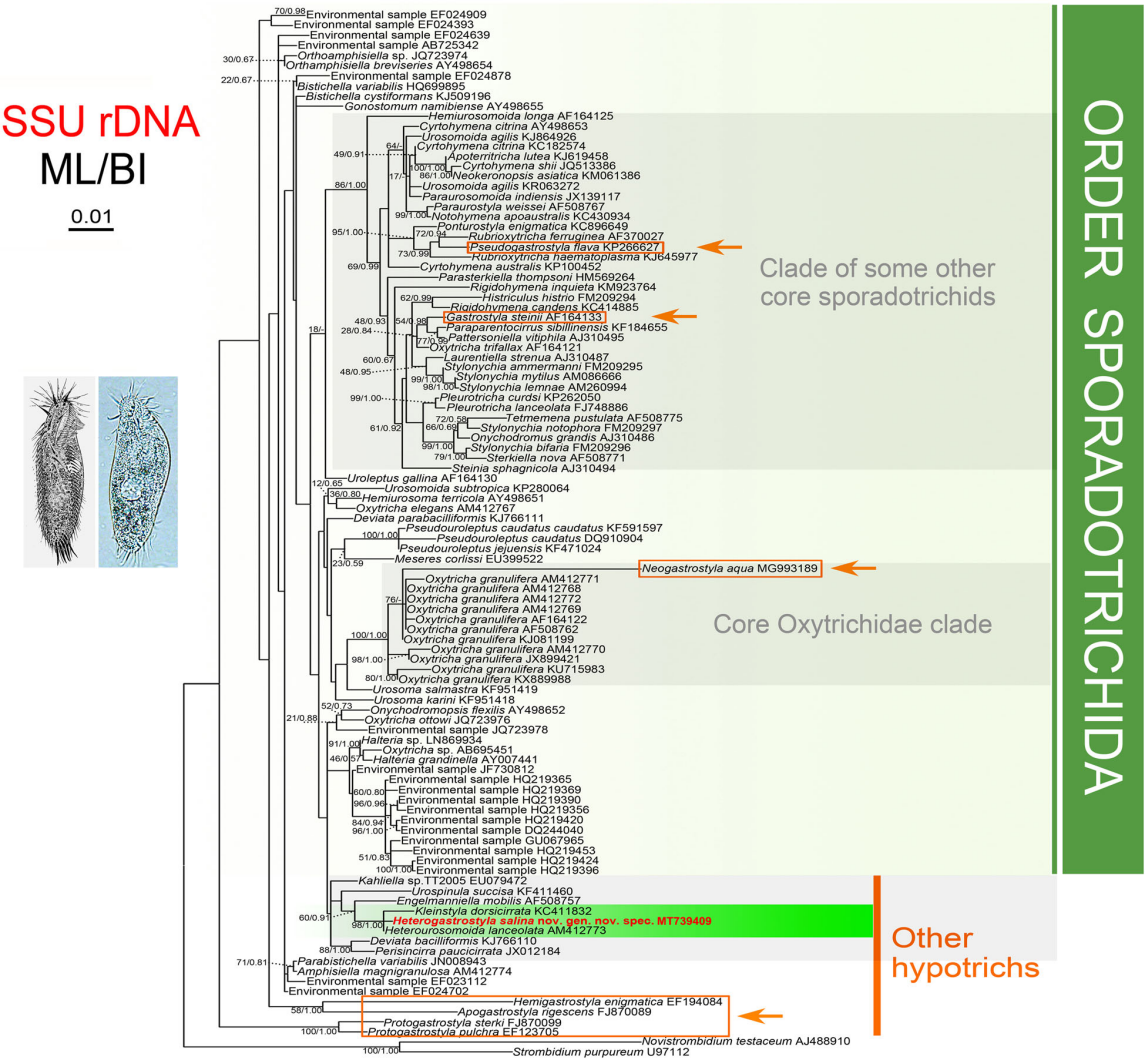
Maximum likelihood (ML) tree inferred from SSU rDNA sequences showing the systematic position of *Heterogastrostyla salina* nov. spec. (bold) and other *Gastrostyla*-like species (frames with arrows). Numbers near nodes are nonparametric bootstrap values for ML and posterior probability values for Bayesian inference (BI). “-” refers to disagreement in topology with the BI tree. All branches are drawn to scale. We have omitted most names of higher taxa because, as in most (all?) other trees, the taxa are non-monophyletic. The scale bar corresponds to 0.01 expected substitutions per site.

The SSU rDNA sequence of *Heterogastrostyla salina* nov. spec. was deposited in the GenBank database with the accession number MT739409. The length and GC content of the SSU rDNA sequence are 1687 bp and 46.00%, respectively. Phylogenetic trees inferred from the SSU rDNA sequences using two different methods, i.e., maximum likelihood (ML) and Bayesian inference (BI), show similar topologies, therefore we present only the ML tree with bootstraps and posterior probabilities from both algorithms (Fig. 1).

*Heterogastrostyla salina* clusters with *Heterourosomoida lanceolata* and *Kleinstyla dorsicirrata* with high support (ML/BI, 98/1.00), differing from them in 8 and 19 nucleotide sites, respectively. Other *Gastrostyla*-like species, distantly related to *H. salina*, fall into several groups: (i) *Hemigastrostyla*, *Apogastrostyla* and *Protogastrostyla* occupy the basal positions within the hypotrich assemblage although with low nodal support; (ii) *Pseudogastrostyla flava* clusters with *Rubrioxytricha ferruginea* with low support (ML/BI, 72/0.94); (iii) *Gastrostyla steinii* nests within the typical oxytrichids; (iv) *Neogastrostyla aqua* falls within a fully supported clade of *Oxytricha granulifera* populations (ML/BI, 100/1.00).

### Cladistics relationship and morphological patterns of *Gastrostyla*-like species (Figs. 2 and 3)

**Fig. 2:**
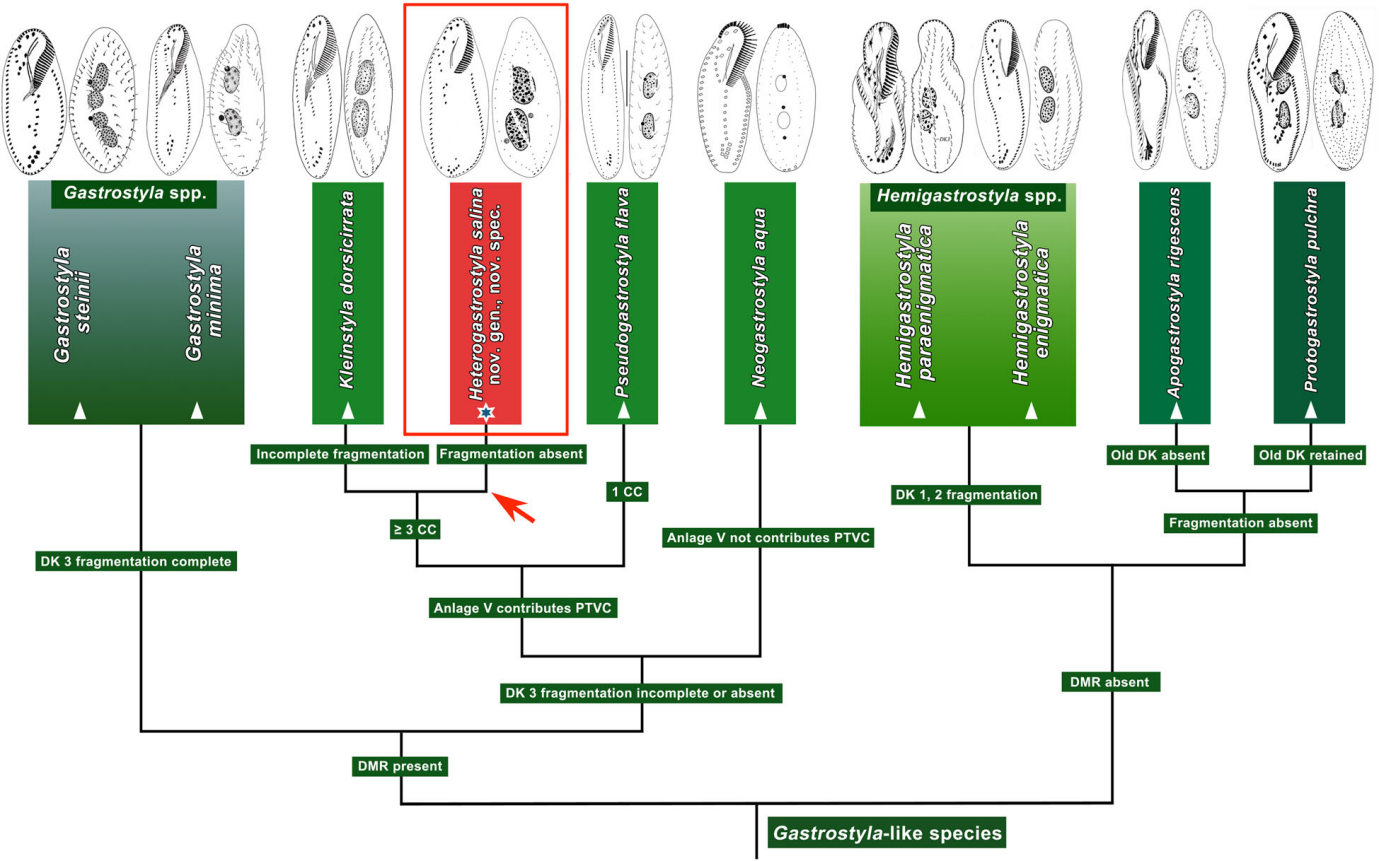
Cladogram of *Gastrostyla*-like species based upon pattern of dorsal kineties, arrow and the red frame mark the *Heterogastrostyla salina* nov. spec. clade. CC, caudal cirri; DK, dorsal kinety; DMR, dorsomarginal row; PTVC, pretransverse ventral cirri.

**Fig. 3:**
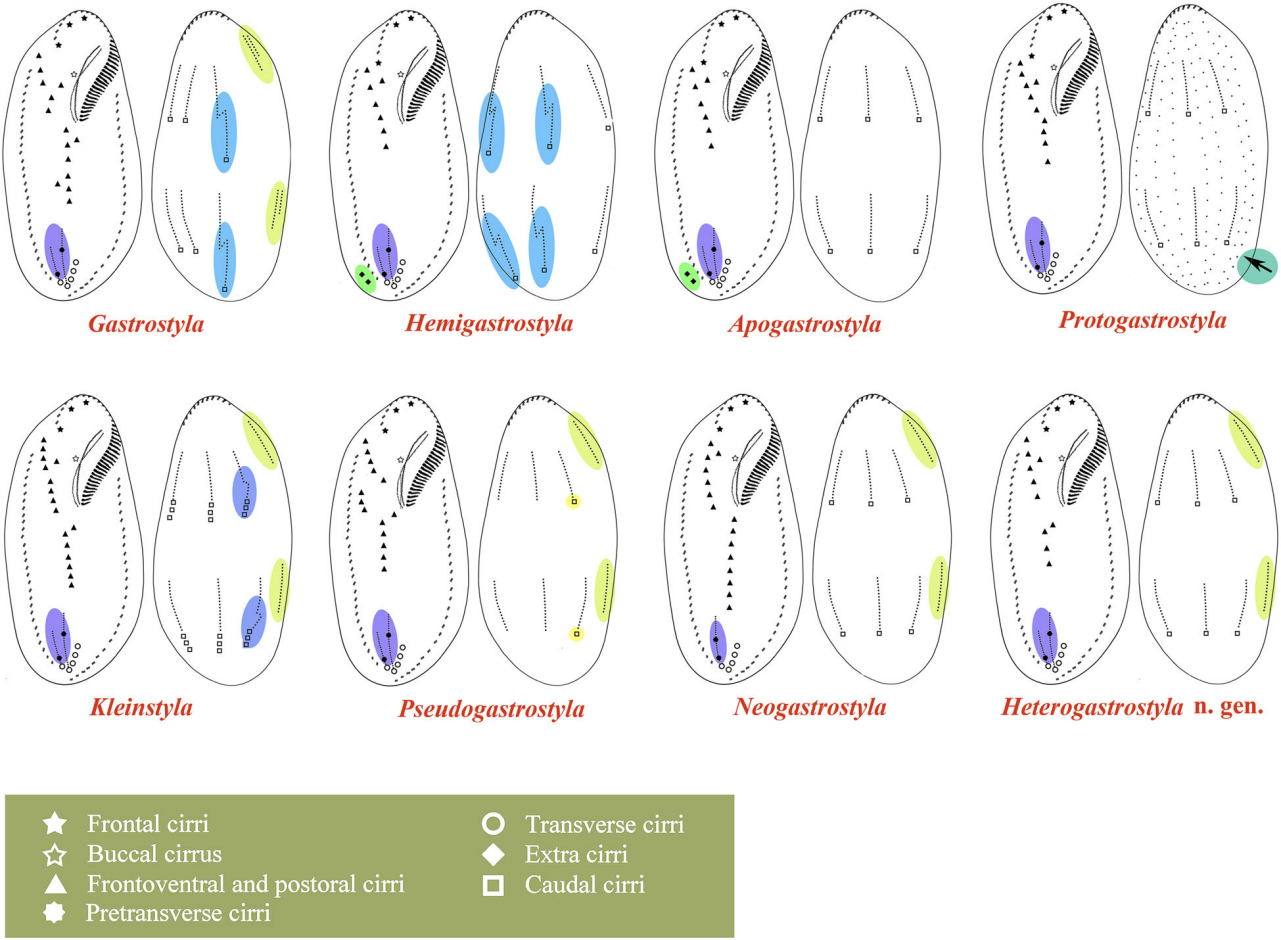
Diagram of the infraciliature, and formation patterns of pretransverse ventral cirri (with dotted lines connecting cirri that develop from the same cirral streaks) and dorsal ciliature of eight genera. Arrow marks the parental kineties which are retained in the daughter cells. Purple, blue and yellow shaded area represent the pretransverse cirri, dorsal kinety pattern and dorsomarginal kineties, respectively.

A cladogram of *Gastrostyla*-like species was constructed based on the presence/absence of dorsomarginal/dorsal fragmentation, the fate of the old dorsal kineties, the number of caudal cirri and whether anlage V contributes to pretransverse ventral cirri (Fig. 2). We also provide illustrations showing the morphology of *Gastrostyla*-like genera for clarity (Fig. 3). *Heterogastrostyla salina*, *Kleinstyla dorsicirrata*, *Pseudogastrostyla flava*, and *Neogastrostyla aqua* form one clade, because they have dorsomarginal rows. Other *Gastrostyla* spp. are separated from this clade due to their incomplete, or the complete absence of, dorsal kinety fragmentation. *Neogastrostyla aqua* is distinguished from *H. salina*, *K. dorsicirrata* and *P. flava* by its anlage V not contributing to pretransverse ventral cirri. *Pseudogastrostyla flava* is distinguished from *H. salina* and *K. dorsicirrata* by the number of caudal cirri. In *H. salina*, the dorsal fragmentation is absent, whereas *K. dorsicirrata* has incomplete fragmentation. As concerns *Gastrostyla*-like taxa without dorsomarginal rows, *Hemigastrostyla* differs from *Apogastrostyla rigescens* and *Protogastrostyla pulchra* in exhibiting multiple dorsal kinety fragmentation, whereas *A. rigescens* and *P. pulchra* are distinguished from each other by the retention/resorption of the parental dorsal kineties.

### Heterogastrostyla nov. gen.

Order Sporadotrichida Fauré-Fremiet, 1961

#### Diagnosis

Body flexible. Undulating membranes slightly curved. More than 18 fronto-ventral-transverse cirri grouped in *Oxytricha*-like pattern. Cirral anlagen V and VI develop pretransverse cirri. One right and one left marginal row. Dorsal ciliature in *Urosomoida*-like pattern: three main dorsal kineties and one dorsomarginal row. Caudal cirri present.

#### Type species

*Heterogastrostyla salina* nov. spec.

#### Etymology

Composite of the Greek adjective *heteros* (different) and the well-known genus name *Gastrostyla*. This indicates that *Heterogastrostyla* has a similar ventral ciliature to *Gastrostyla* but differs in the dorsal side. Feminine gender.

### Heterogastrostyla salina nov. spec.

#### Diagnosis

Size in vivo 100–120 × 30–45 μm, outline in ventral view elliptical. Two macronuclear nodules, two micronuclei. Contractile vacuole slightly ahead of mid-body. Adoral zone composed of 25–31 membranelles. 21–24 fronto-ventral-transverse cirri, some frontoventral and postoral ventral cirri form a more or less continuous row. Left and right marginal row composed of 27–37 and 21–31 cirri, respectively. Three bipolar dorsal kineties with one short dorsomarginal kinety in *Urosomoida-*like pattern. Three caudal cirri. Saline soil habitat.

#### Type locality

**Fig. 4:**
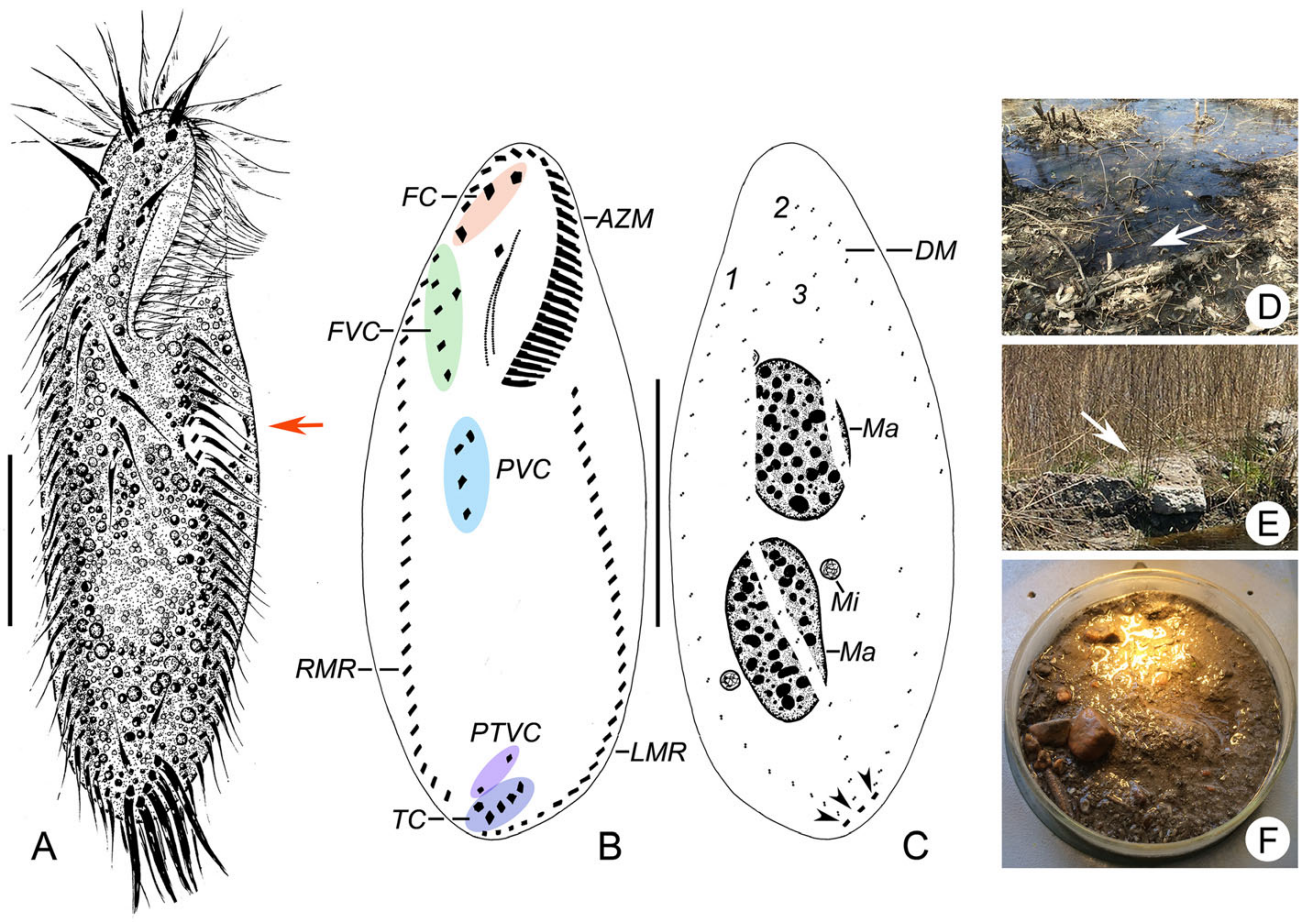
**a-c** Morphology and infraciliature and of *Heterogastrostyla salina* nov. spec. from life (**a**) and after protargol staining (**b**, **c**). **a** Ventral view of a typical individual, arrow marks the contractile vacuole. **b**, **c** Ventral (**b**) and dorsal (**c**) view of the same individual, arrowheads in (**c**) depict the caudal cirri. **d**, **e** Surroundings of the sampling sites, arrows indicate where the soil samples were collected. **f** Photograph showing the raw culture in a non-flooded Petri dish. AZM, adoral zone of membranelles; DM, dorsomarginal kinety; FC, frontal cirri; FVC, frontoventral cirri; LMR, left marginal row; Ma, macronuclear nodules; Mi, micronuclei; PVC, postoral ventral cirri; PTVC, pretransverse ventral cirri; RMR, right marginal row; TC, transverse cirri. 1–3, dorsal kineties 1–3. Scale bars = 30 μm (**a**); 40 μm (**b**, **c**).

Saline soil from the Longfeng Wetland Nature Reserve, Daqing, northern China (Fig. 4 d–f; lat. 46°35′30″N, long. 125°13′08″E; for details, see Material and Methods).

#### Etymology

The species-group name *salina* refers to the saline habitat where the type specimen was discovered.

#### Type slides

**Fig. 5:**
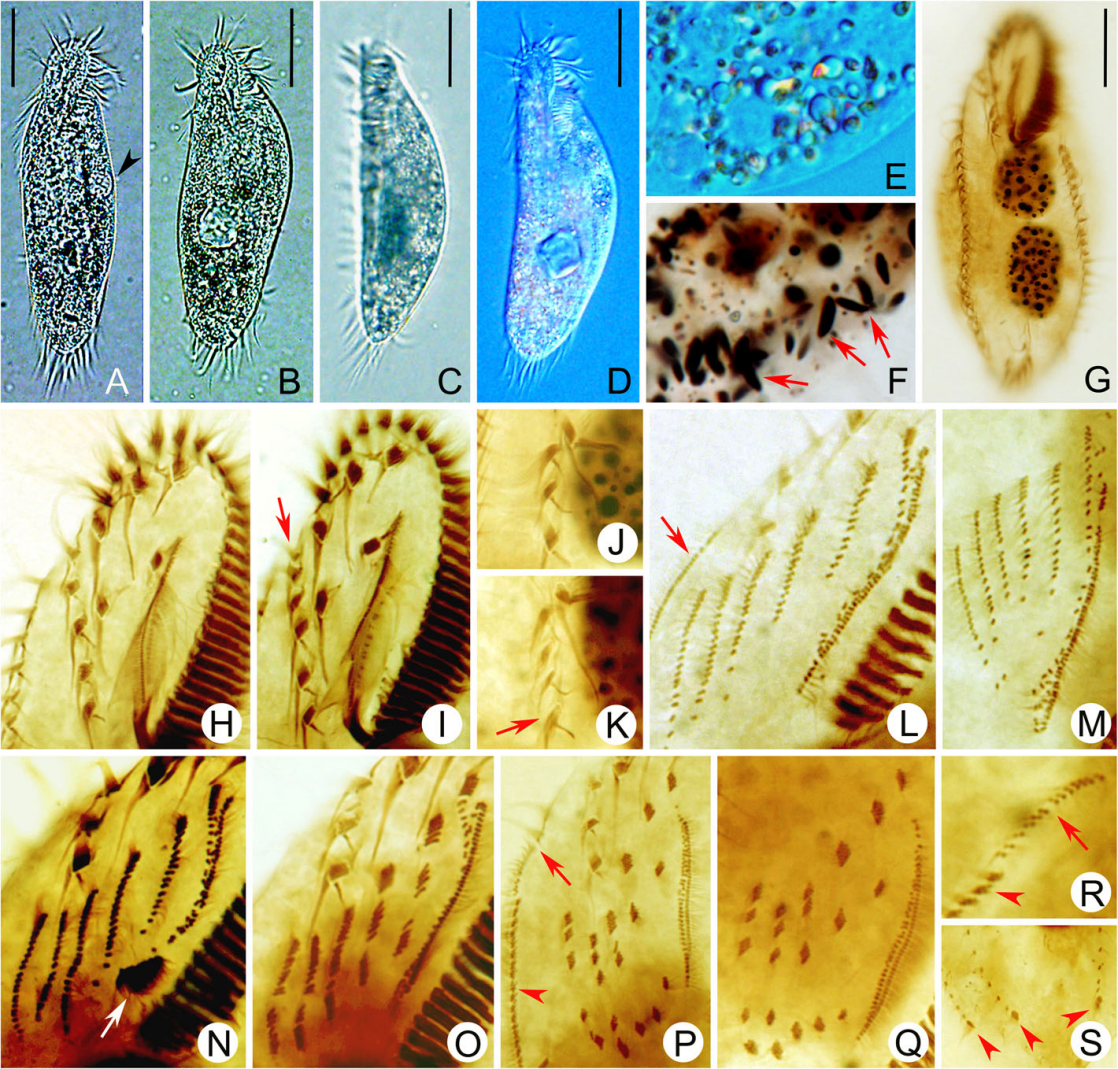
**a-s** Photomicrographs of *Heterogastrostyla salina* nov. spec. from life (**a**–**e**) and after protargol staining (**f**–**s**). **a–d** Different body shapes, arrowhead in (**a**) indicates the contractile vacuole. **e** Cytoplasm containing numerous lipid droplets and refractive crystals. **f** Showing the spindle-shaped extrusomes (arrows) after protargol staining. **g** Ventral view of the holotype specimen. **h**, **i** Ventral view to show the frontoventral ciliature, arrow depicts the additional frontoventral cirri. **j**, **k** Ventral view to show the postoral ventral ciliature, arrow indicates the additional postoral ventral cirri. **l**, **n**–**p**, **r** Development ventral development in the proter, arrow in (**l**) and arrowhead in (**r**) indicate the anlage for the right marginal row; arrow in (**n**) depicts the small patch of densely distributed kinetids posterior to the frontoventral anlage I; arrows in (**p**) and (**r**) denote the dorsomarginal anlage (dikinetidal row) that originates from the anterior of the right marginal anlage. **m**, **q** Development fronto-ventral-transverse anlagen in the opisthe. **s** Dorsal view of a late divider, arrowheads show the caudal cirri. Scale bars = 30 μm (**a**–**e**).

The protargol-stained slide with the holotype specimen (Figs. 4 b, c and 5 g) circled in ink is deposited in the Natural History of Museum, London, UK (registration number NHMUK2020.4.4.1). One protargol slide with paratype specimens are deposited in the Laboratory of Protozoology, Ocean University of China (OUC, registration number: Leo2015041601).

#### ZooBank registration

Registration number of the present work: lsid:zoobank.org:pub:B0946886-C083-421B-8BD3-35D261200B79

### Morphology of *Heterogastrostyla salina* nov. spec. (Figs. 4 a–c and 5 a–k, Table 1)

Body size 100–120 × 30–45 μm in vivo (*n =* 12), usually 120 × 40 μm; in protargol preparations 110 × 45 μm on average. Specimens widened during protargol preparation procedures (Table 1); length to width ratio about 3: 1 in vivo (Figs. 4 a and 5 a–d). Cell outline long elliptical or lanceolate, rounded at both ends with anterior portion sometimes slightly narrowed (Figs. 4 b and 5 b); cell flexible and slightly contractile. Body dorsoventrally flattened about 1.5:1, dorsal side slightly convex, ventral side slightly concave (Fig. 5 c). Nuclear apparatus located along, or slightly left of, cell midline, composed of two ellipsoidal macronuclear nodules and one to six, on average two, micronuclei attached, or near to the macronuclear nodules; macronuclear nodules about 25 × 15 μm in size (in protargol preparations), micronuclei about 4 μm across (Fig. 4 c; Table 1). Cortical granules not observed. In protargol-impregnated specimens, extruded spindle-shaped extrusomes, 2–3 × 1–1.5 μm, were observed in marginal region of cortex (Fig. 5 f). Cytoplasm colourless to greyish, containing numerous lipid droplets (ca. 2–3 μm across) and refractive crystals (1–6 μm across) that render cell opaque and dark at low magnification (Fig. 5 c–e). One contractile vacuole about 12 μm across, positioned slightly ahead of mid-body, near left margin (Figs. 4 a and 5 a). Locomotion mainly by slow to fast crawling on substrate; in cultures, cells usually aggregate around rice grains or bottom detritus.

**Table 1:** Morphometric characterisation of *Heterogastrostyla salina* nov. spec

Character^**a**^	Min	Max	Mean	M	SD	CV	n
Body length	95	130	111.5	115	9.2	8	25
Body width	35	55	44.8	45	4.5	10	25
Body length: width ratio	2.1	3.0	2.5	2.5	0.3	10.1	25
Paroral, length	20	25	23.6	25	2.3	9.2	25
Endoral, length	20	25	23.8	25	2.2	8.7	25
Adoral zone of membranelles, length	30	45	38.3	40	3.9	9.9	25
Adoral zone of membranelles, length: body length ratio	0.26	0.47	0.34	0.35	0.04	11.6	25
Adoral membranes, number	25	31	27.8	28	1.8	6.3	25
Frontal cirri, number	3	3	3.0	3	0.0	0.0	25
Buccal cirri, number	1	1	1.0	1	1.0	1.0	25
Frontoventral cirri, number	6	8	6.3	6	0.6	9.3	25
Postoral ventral cirri, number	4	5	4.0	4	0.2	5.0	25
Pretransverse ventral cirri, number	2	2	2.0	2	0.0	0.0	25
Transverse cirri, number	4	5	4.9	5	0.3	5.5	25
Right marginal cirri, number	21	31	27.0	27	2.3	8.7	25
Left marginal cirri, number	27	37	32.6	33	2.1	6.3	25
Dorsal kineties, number	3	3	3.0	3	0.0	0.0	25
Dorsomarginal row, number	1	1	1.0	1	1.0	1.0	25
Caudal cirri, number	3	3	3.0	3	0.0	0.0	25
Macronuclear nodules, number	2	2	2.0	2	0.0	0.0	25
Micronuclei, number	1	6	2.0	2	1.1	56.6	25
Anterior macronuclear nodule, length	20	30	23.4	24	3.0	12.6	25
Anterior macronuclear nodule, width	10	16	14.9	15	1.5	10.2	25
Micronuclei, length	3	5	4.1	4	0.9	21.7	25
Micronuclei, width	3	5	4.1	4	0.9	21.7	25

^a^All data are based on protargol-stained specimens.

*CV* coefficient of variation in %, *M* median, *Max* maximum, *Mean* arithmetic mean, *Min* minimum, *n* number of cells measured, *SD* standard deviation. The length measurement are in microns.

Adoral zone about 40 μm long, composed of 25–31 membranelles, occupying ca. 30% of body length in vivo, and about 35% in protargol preparations; cilia of distal membranelles 15–20 μm long; cilia of proximal membranelles 10–15 μm long. Undulating membranes almost straight and in *Oxytricha*-like pattern, paroral and endoral almost equal in length, about 25 μm long. Three enlarged frontal cirri, cilia of which are ca. 20 μm long. One buccal cirrus, ca. 15 μm long, located adjacent to anterior end of paroral. Six (rarely seven or eight) frontoventral cirri (Figs. 4 b and 5 h, i), cilia of which are 12–15 μm long. Four (rarely five) postoral ventral cirri (Figs. 4 b and 5 j, k), cilia of which are 12–15 μm long. All frontoventral and postoral ventral cirri (except of cirri III/2 and IV/2) form a more or less continuous row (Figs. 4 b and 5 h–k). Left and right marginal row composed of 27–37 and 21–31 cirri, respectively, cilia of which are 12–15 μm long; left row J-shaped, terminates at posterior end of cell, behind the rearmost transverse cirrus; right row commences at about level of second frontoventral cirrus and terminates at about level of lower pretransverse cirrus. Two pretransverse cirri. Five enlarged transverse cirri in J-shaped pattern (Fig. 4 b). Dorsal kineties in typical *Urosomoida*-like pattern, i.e. three dorsal kineties, each bearing a caudal cirrus at posterior end, and one dorsomarginal row terminating at about mid-body; dorsal kinety 1 (leftmost) usually slightly to distinctly shortened anteriorly (Fig. 4 c).

### Ontogenesis of *Heterogastrostyla salina* nov. spec. (Figs. 5 l–s, 6 a–j and 7 a, b)

**Fig. 6:**
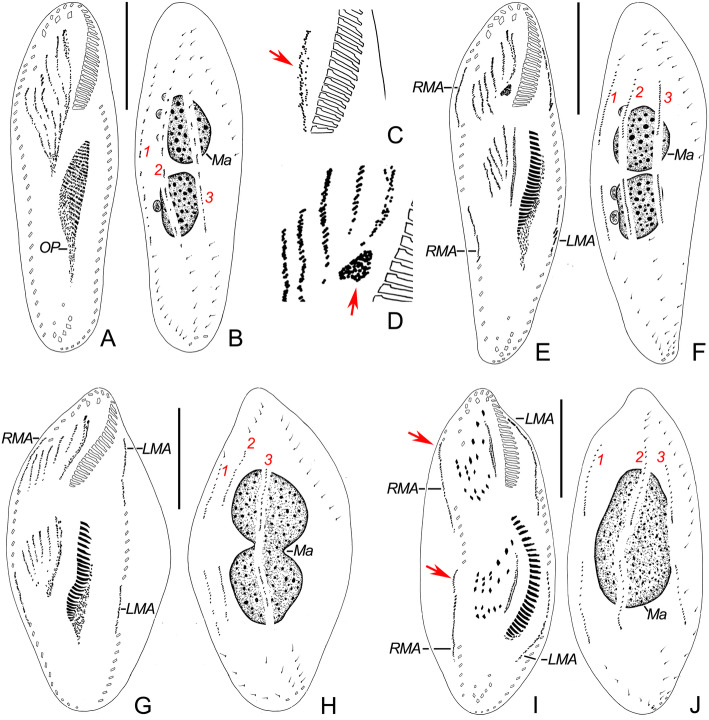
**a-j** Early and middle stages of morphogenesis in *Heterogastrostyla salina* nov. spec. after protargol staining. **a**, **e**, **g**, **i** Ventral views to show the development of oral primordium, fronto-ventral-transverse anlagen and marginal rows anlagen, arrows in (**i**) indicate dorsomarginal anlagen (dikinetidal row) that originate from the anterior of right marginal anlage. **c** Showing the resorption of parental undulating membranes (arrow) in the proter as depicted in (**a**). **d** Magnified view of the fronto-ventral-transverse anlagen as shown in (**e**), arrow indicates the small patch of densely distributed kinetids posterior to the frontoventral anlage I in the proter. **b**, **f**, **h**, **j** Dorsal views to show the development of dorsal kineties and nuclear apparatus. LMA, anlage for the left marginal row; Ma, macronuclear nodules; OP, oral primordium; RMA, anlage for the right marginal row; 1−3, dorsal kineties anlagen 1−3. Scale bars = 40 μm **a**−**j**.

The earliest stage observed had six long primary fronto-ventral-transverse (FVT) anlagen with a differentiating oral primordium in the opisthe (Fig. 6 a).

**Fig. 7:**
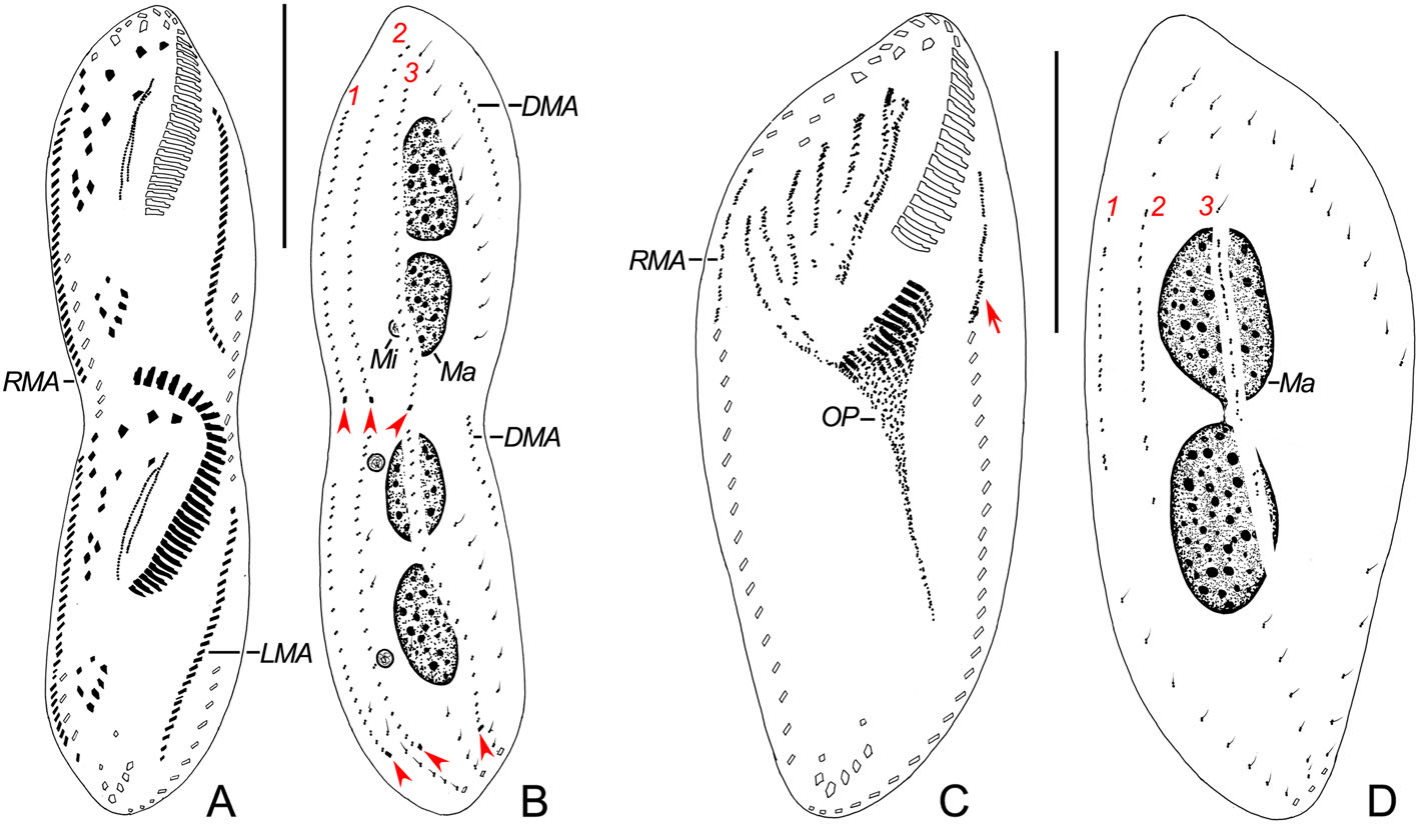
**a-d** Late stage of morphogenesis (**a**, **b**) and middle stage of reorganization (**c**, **d**) in *Heterogastrostyla salina* nov. spec. after protargol staining. **a**, **c** Ventral views to show the development of the oral primordium, fronto-ventral-transverse anlagen and marginal rows anlagen, arrow in (**c**) depicts the anlage for the left marginal row. **b**, **d** Dorsal views to show the development of dorsal kineties and nuclear apparatus, arrowheads in (**b**) indicate the newly differentiated caudal cirri. DMA, dorsomarginal anlage; Ma, macronuclear nodules; Mi, micronuclei; OP, oral primordium; RMA, anlage for the right marginal row; 1−3, dorsal kineties anlagen 1−3. Scale bars = 50 μm (**a**, **b**); 40 μm (**c**, **d**).

#### Stomatogenesis

In the opisthe, the formation of membranelles commences left of the anterior end of the oral primordium (Fig. 6 a). As the formation of adoral membranelles proceeds posteriad, the undulating membranes anlage (= FVT-anlage I) contributes the leftmost frontal cirrus and splits longitudinally into two streaks from which the endoral and paroral are formed (Figs. 5 l and 6 e, g, i).

In the proter, the parental undulating membranes gradually dedifferentiate into an undulating-membrane anlage. The differentiation of the undulating membranes anlage follows a similar pattern to that in the opisthe (Fig. 6 c, e, g, i). Interestingly, on one middle stage specimen, we found a small patch of densely distributed kinetids posterior to frontoventral anlage I in the proter (Figs. 5 n and 6 d), but this small patch disappeared in the next stage (Fig. 6 g). We deduce that it is a remnant of the FVT-anlagen. The parental adoral zone of membranelles is retained intact during the morphogenetic process (Figs. 6 a, e, g, i and 7 a).

#### Development of frontoventral ciliature

The FVT-anlagen II-VI develop as primary primordia and then divide into two groups transversely, one for each daughter cell (Figs. 5 l–n and 6 a, e, g). Subsequently, anlagen I–VI of each group segregate new cirri in the normal pattern: 1:3:3:4:5:5 (Figs. 5 o q and 6 i). After migration and differentiation, three frontal, one buccal, six to eight frontoventral, four or five postoral ventral, two pretransverse and five transverse cirri are formed. The origination of ventral ciliature is as follows: (i) the leftmost front frontal cirrus comes from anlage I; (ii) the middle frontal cirrus and buccal cirrus come from anlage II; (iii) the rightmost frontal cirrus originates from anlage III; (iv) the six frontoventral cirri come from anlage III (×1), anlage IV (×2) and anlage V (×3); (v) the four postoral ventral cirri come from anlage IV (×1) and anlage V (×3); the two pretransverse cirri come from anlage V (×1) and anlage VI (×1); and the five transverse cirri derive from the posterior end of anlagen II–IV, respectively (Figs. 6 i and 7 a).

#### Marginal rows

In each divider, the marginal rows anlagen develop intrakinetally. These anlagen then increase in size by adding basal bodies on the right side of the parental structure (Fig. 6 e). Meanwhile, the parental rows are gradually resorbed (Fig. 6 g, i).

#### Dorsal kineties

In the earliest stage, several patches of the dorsal-kinety (DK) anlagen appeared intrakinetally in the middle of each old structure without a clear separation for the proter and the opisthe (Fig. 6 b); Whether the DK-anlagen are primary primordia is not clear, however, since their early development is not known. Dorsal morphogenesis proceeds in *Urosomoida*-like pattern, i.e. the new dorsal kineties develop intrakinetally as three anlagen each in the proter and opisthe without fragmentation, and each dorsal kinety produces one caudal cirrus in the late stage (Figs. 5 s, 6 b, f, h, j and 7 b). It is noteworthy that a short dikinetid-row appears anterior of the right marginal anlagen, more or less distinctly separated from the right marginal anlagen (Figs. 5 p, r and 6 i). This is the dorsomarginal kinety anlage. It is unclear whether the dorsomarginal kinety anlage derives from the anterior portion of the right marginal anlage and later moves to the dorsal side.

#### Division of nuclear apparatus

The nuclear apparatus divides in the usual way, i.e., the two macronuclear nodules fuse to form a single mass during the mid-divisional stage which then divide twice prior to cytokinesis (Figs. 6 b, f, h, j and 7 b).

### Physiological reorganization

Only one early stage of physiological reorganization was observed (Fig. 7 c, d), which indicated that the early process of cortical development in reorganizers is similar to morphogenesis.

## Discussion

### Phylogenetic position of the new genus *Heterogastrostyla* and related taxa

The present phylogenetic analyses show that *Heterogastrostyla* nov. gen. is most closely related to *Heterourosomoida* and *Kleinstyla*. However, the systematic position of this group is far from being resolved, as indicated by the variable statistical support in the SSU rDNA tree (Fig. 1).

The grouping of *Heterogastrostyla*, *Heterourosomoida*, and *Kleinstyla* was supported by their morphological similarities in that all these three genera exhibit deviation from the typical oxytrichid fragmentation of dorsal kinety 3. The former two genera share the same *Urosomoida*-like pattern in which fragmentation of dorsal kinety 3 is lost, whereas *Kleinstyla* exhibits incomplete fragmentation of dorsal kinety 3 [18]. *Neogastrostyla aqua*, resembles *H. salina* in terms of the dorsal ciliary pattern, however, they are not closely related in the SSU rDNA tree as *N. aqua* nests robustly within the *Oxytricha granulifera* clade. Similarly, *Gastrostyla* is distinctively placed within the oxytrichid clade, which is consistent with assertion of Wirnsberger et al. (1986) that *G. steinii* is a stylonychine oxytrichid [31]. Other *Gastrostyla*-like genera, i.e., *Pseudogastrostyla*, *Apogastrostyla*, *Protogastrostyla*, and *Hemigastrostyla*, are consistently placed outside the oxytrichid clade, as shown in the previous studies [17, 23].

The cladogram based on the dorsal ciliary pattern (Fig. 2) of *Gastrostyla*-like species is broadly consistent with the molecular tree (Fig. 1). With the presence of the dorsomarginal row, *Heterogastrostyla salina* shows a close relationship with *Kleinstyla dorsicirrata* and *Pseudogastrostyla flava*. Together with *Gastrostyla* spp., they are closely related to oxytrichids, whereas *Apogastrostyla*-*Protogastrostyla*-*Hemigastrostyla* are distinctly separated from the oxytrichid clade since they lack a dorsomarginal row. The presence/absence of dorsomarginal kineties plays a significant role in the classification of hypotrichs, supporting the Dorsomarginalia hypothesis [32].

### Establishment of the new genus

In possessing more than 18 fronto-ventral-transverse cirri that form a continuous, slightly oblique row, *Heterogastrostyla* nov. gen. is similar to the following genera: *Neogastrostyla* Kaur et al., 2019 [20], *Gastrostyla* Engelmann, 1862 [21], *Kleinstyla* Foissner et al., 2002 [22], *Pseudogastrostyla* Fan et al., 2015 [24], *Hemigastrostyla* Song and Wilbert, 1997 [19], *Apogastrostyla* Li et al., 2010 [17] and *Protogastrostyla* Gong et al., 2007 [23]. The most distinct feature that separates *Heterogastrostyla* from related genera is its *Urosomoida-*like dorsal ciliature pattern, i.e., fragmentation of dorsal kinety anlage 3 is lost during ontogenesis. Based on this, *Neogastrostyla* closely resembles *Heterogastrostyla*. However, the former can be distinguished from the latter by cirral anlage V not contributing a pretransverse ventral cirrus, i.e. both pretransverse cirri develop from anlage VI (vs. cirral anlagen V and VI each generates a pretransverse ventral cirrus in *Heterogastrostyla*). Due to these morphogenetic differences, a new genus is established.

### Morphological comparison of *Heterogastrostyla salina* nov. spec. with related *Gastrostyla*-like taxa

*Heterogastrostyla salina* belongs to the group of *Gastrostyla*-like taxa which share a similar ventral cirral pattern: (i) frontoventral cirri derived from anlagen IV–VI form a more or less continuous, slightly oblique row, and (ii) fronto-ventral-transverse cirri either retain the typical pattern of 18 FVT-cirri or number more than 18 FVT-cirri. We therefore compare our new form with eight typical *Gastrostyla*-like species, namely, *Neogastrostyla aqua* Kaur et al., 2019, *Gastrostyla steinii* Engelmann, 1862, *G. minima* Hemberger, 1985, *Kleinstyla dorsicirrata* (Foissner, 1982) Foissner et al., 2002, *Pseudogastrostyla flava* Fan et al., 2015, *Hemigastrostyla paraenigmatica* Shao et al., 2011, *H. enigmatica* (Dragesco and Dragesco-Kernéis, 1986) Song and Wilbert, 1997, *Apogastrostyla rigescens* (Kahl, 1932) Li et al., 2010, and *Protogastrostyla pulchra* (Perejaslawyewa, 1886) Gong et al., 2007.

*Neogastrostyla aqua* can be easily distinguished from *Heterogastrostyla salina* by the number of dorsomarginal kineties (two vs. one), frontoventral cirri (that is, cirri in the anterior portion of frontoventral row in Kaur et al. 2019) (5 or 6 vs. 6–8) and postoral ventral cirri (that is, postoral ventral cirri and cirri in the posterior portion of frontoventral row in Kaur et al. 2019) (8 or 9 vs. 4 or 5) [20].

*Gastrostyla steinii*, the type species of the genus *Gastrostyla*, can be easily distinguished from *Heterogastrostyla salina* by: (i) the number of macronuclear nodules (four vs. two); (ii) a higher and variable number of fronto-ventral-transverse cirri (27–32 vs. 21); (iii) the segregation pattern of fronto-ventral-transverse cirri from anlagen I–VI (1:2:3:3:11–13:7–10 vs. 1:3:3:4:5:5); and (iv) the dorsal kinety pattern (*Oxytricha*-like pattern vs. *Urosomoida-*like pattern) [16, 33].

*Gastrostyla minima* differs from *Heterogastrostyla salina* as follows: (i) cortical granules present (vs. absent); (ii) dorsal kineties in an *Oxytricha-*like (vs. *Urosomoida-*like) pattern; (iii) some cirri greatly reduced in size and/or slightly out of line (vs. cirri of uniform size and aligned regularly), and; (iv) the total number of fronto-ventral-transverse cirri (22–31 vs. 21) [16, 34, 35].

*Heterogastrostyla salina* can be distinguished from *Kleinstyla dorsicirrata* by: (i) the number of caudal cirri (three, one each at the posterior end of kineties 1–3 vs. nine on average, with 3–6, 2–4, 1–3 at the end of kineties 1–3, respectively); (ii) dorsal kineties in a *Urosomoida-*like (vs. *Oxytricha-*like) pattern; (iii) the segregation pattern of fronto-ventral-transverse cirri from anlagen I–VI (1:3:3:4:5:5 vs. 1:3:3:4–7:6–10:6–9), and; (iv) the total number of fronto-ventral-transverse cirri (21 vs. 28) [16, 33, 35].

*Pseudogastrostyla flava* can be easily distinguished from *Heterogastrostyla salina* by: (i) the number of caudal cirri (one vs. three); (ii) cortical granules (present vs. absent), and; (iii) biotope (brackish water vs. terrestrial) [24].

*Heterogastrostyla salina* can be distinguished from *Hemigastrostyla paraenigmatica* by: (i) the shape of the anterior portion of the cell (not cephalized vs. cephalized); (ii) the extra cirri (absent vs. present); (iii) cortical granules (absent vs. present); (iv) the length of the distal portion of the adoral zone (not extending far posteriorly vs. extending far posteriorly); (v) the number of adoral membranelles (28 vs 42); (vi) dorsal kineties in a *Urosomoida-*like (vs. *Hemigastrostyla-*like) pattern; (vii) the number of dorsal kineties (three vs. five), and; (viii) the total number of fronto-ventral-transverse cirri (21 vs. 18) [16, 19, 36, 37].

*Heterogastrostyla salina* differs from *Hemigastrostyla enigmatica* by: (i) dorsal kineties in a *Urosomoida-*like (vs. *Hemigastrostyla-*like) pattern; (ii) the number of dorsal kineties (three vs. five); (iii) the extra cirri (absent vs. present); (iv) the body size (110 × 45 μm vs. 142 × 63 μm in protargol preparation); (v) the length of the distal portion of adoral zone (not extending far posteriorly vs. extending far posteriorly); (vi) the number of adoral membranelles (28 vs 50); (vii) the length of the adoral zone (38 μm vs. 57 μm), and; (viii) the total number of fronto-ventral-transverse cirri (21 vs. 18) [38].

*Heterogastrostyla salina* can be separated from *Apogastrostyla rigescens* by: (i) the shape of the anterior portion of the cell (not cephalized vs. cephalized); (ii) the pattern of undulating membranes (*Oxytricha*-like vs. *Stylonychia*-like); (iii) the length of the distal portion of adoral zone (not extending far posteriorly vs. extending far posteriorly); (iv) the extra cirri (absent vs. present); (v) dorsal kineties in a *Urosomoida-*like (vs. *Gonostomum-*like) pattern; (vi) cortical granules (absent vs. present); (vii) ring-shaped structures (absent vs. present); (viii) the body size (110 × 45 μm vs. 150 × 42 μm in protargol preparation); (ix) the number of adoral membranelles (28 vs 43); (vi) the length of the adoral zone (38 μm vs. 61 μm); (vii) the total number of fronto-ventral-transverse cirri (21 vs. 18), and; (viii) biotope (terrestrial vs. marine) [17, 29].

*Protogastrostyla pulchra* differs from *Heterogastrostyla salina* by: (i) the fate of the parental dorsal kineties (retained vs. resorbed); (ii) the number of dorsal kineties (9–11 vs. three); (iii) the number of adoral membranelles (54 vs 28); (iv) the length of the distal portion of adoral zone (extending far posteriorly vs. not extending far posteriorly); (v) the position of the transverse cirri (distinctly displaced anteriad vs. close to rear body margin); (vi) cortical granules (present vs. absent); (vii) the body size (153 × 72 μm vs. 110 × 45 μm, in protargol preparations); (viii) the course of the right and left marginal rows (overlapping at rear end of cell vs. not overlapping), and; (ix) biotope (marine vs. terrestrial) [16, 23, 39–41].

*Heterogastrostyla salina* resembles *Urosomoida*, *Paraurosomoida*, *Hemiurosomoida*, and *Heterourosomoida* in terms of its dorsal ciliature, i.e., fragmentation of dorsal kinety 3 is lost. However, *H. salina* can be distinguished from these taxa by its *Gastrostyla*-like ventral cirral pattern.

### Morphogenesis of *Heterogastrostyla salina* nov. spec.

Morphogenetic characteristics of the new species basically correspond with that of *Neogastrostyla aqua* Kaur et al., 2019, except for development of the pretransverse ventral cirri [20]. In the latter, all cirri (except for a single transverse cirrus) generated from cirral anlage V, move anteriad to form the postoral ventral cirri, that is, cirral anlage V does not develop any pretransverse cirri. However, in *H. salina*, some cirri generated from cirral anlage V move anteriad to form postoral ventral cirri, while others move posteriad to form a pretransverse and a transverse cirrus.

Although morphogenesis of the new species closely resembles that of *Gastrostyla* spp., it differs in that: (1) fronto-ventral-transverse cirral anlagen are formed from primary primordia (vs. in secondary primordia), and; (2) the dorsal kineties anlagen are in a *Urosomoida*-like (vs. an *Oxytricha*-like) pattern [4, 33, 42]

A comparison of ontogenesis of *Gastrostyla*-like species is summarized in Table 2. The ventral development of *Heterogastrostyla salina* proceeds basically as in *Hemigastrostyla*, *Apogastrostyla*, and *Protogastrostyla*. Specifically, six primary FVT-anlagen generate an increased number (>18) of fronto-ventral-transverse cirri, with frontoventral cirri not regularly grouped but arranged in a more or less continuous, slightly oblique (frontoventral) row. *Heterogastrostyla salina*, however, differs significantly from the above mentioned three genera in: (i) the fate of the parental adoral membranelles in the proter (completely retained vs. only apical part of old adoral zone retained, combining the newly built membranelles formed from the proter’s oral primordium), and; (ii) the dorsal development (*Urosomoida-*like pattern vs. *Gonostomum*-like pattern or *Hemigastrostyla*-like pattern). It should be noted that in *Protogastrostyla*, the dorsal kinety anlagen are unique since the primary primordia and old dorsal kineties are retained, resulting in a higher number (9–11) of dorsal kineties [28].

**Table 2:** Morphogenesis comparisons of *Gastrostyla-like* species

Character^a^	*Heterogastrostyla salina* nov. gen., nov. spec.	*Neogastrostyla aqua*	*Gastrostyla* spp*.*	*Hemigastrostyla paraenigmatica*	*Hemigastrostyla enigmatica*	*Apogastrostyla rigescens*	*Protogastrostyla pulchra*
Parental AZM	Completely retained	Completely retained	Completely retained	Only apical part retained	Only apical part retained	Only apical part retained	Only apical part retained
FVTA	Primary primordia	Primary primordia?	Secondary primordia	Primary primordia	Primary primordia	Primary primordia	Primary primordia
Anlage V contribute PTVC	Yes	No	Yes	Yes	Yes	Yes	Yes
RMA	Intrakinetally	Intrakinetally	Intrakinetally	De novo	De novo	De novo	De novo
LMA	Intrakinetally	Intrakinetally	Intrakinetally	Intrakinetally	De novo	Intrakinetally	Intrakinetally in proter and/or de novo in opisthe (?)
RMA anterior part differentiated into paired basal bodies, which then become dorsomarginal row	Yes	No	No	Yes	No	No	Yes
Formation of extra cirri	No	No	No	Yes	Yes	Yes	No
DKA	Likely primary primordia	Secondary primordia	Secondary primordia	Likely primary primordia	Secondary primordia	Likely primary primordia	Primary primordia
Dorsal pattern	*Urosomoida*-like pattern	*Urosomoida*-like pattern	*Oxytricha*-like pattern	*Hemigastrostyla*-like pattern	*Hemigastrostyla*-like pattern	*Gonostomum*-like pattern	*Gonostomum*-like pattern
Parental DK	Completely resorbed	Completely resorbed	Completely resorbed	Completely resorbed	Completely resorbed	Completely resorbed	Retained
Data source	**present work**	Kaur et al. (2019) [20]	Foissner (1982) [33]	Song and Wilbert (1997) [19]	Shao et al. (2013) [38]	Li et al. (2010) [17]	Hu and Song (2000) [39]

^a^Data are based on protargol-stained specimens. Measurements in μm.

Abbreviations: *AZM* adoral zone of membranelles, *DK* dorsal kineties, *DKA* dorsal kinety anlagen, *FVTA* fronto-ventral-transverse anlagen, *LMA* left marginal anlage, *PTVC* pretransverse ventral cirri, *RMA* right marginal anlage.

## Conclusions

Similar to the CEUU-hypothesis in urostylids and uroleptids (Foissner et al., 2004), we speculate that the shared ventral cirral patterns of *Gastrostyla*-like species might result from the convergent evolution from four major groups: (i) true oxytrichids with both a dorsomarginal row and complete fragmentation of dorsal kinety 3 (e.g., *Gastrostyla steinii*); (ii) those with a dorsomarginal row but without, or with incomplete, dorsal fragmentation (e.g., *Heterogastrostyla* nov. gen., *Kleinstyla*, and *Pseudogastrostyla*); (iii) those without a dorsomarginal row but with multiple fragmentation of dorsal kineties 1 and 2 (e.g., *Hemigastrostyla*); and (iv) those in which both the dorsomarginal row and dorsal kinety fragmentation are absent (e.g., *Protogastrostyla* and *Apogastrostyla*). We assume that the “*Gastrostyla*-like ventral cirral pattern” evolved at least twice independently in the above-mentioned groups. Since the ventral ciliature is linked with motility, foraging and food uptake, the evolutionary pressure on the ventral ciliature is much stronger than on the dorsal ciliature, which is possibly sensoric and therefore is more conservative [25]. This might explain why the *Gastrostyla*-like species are scattered throughout the SSU rDNA tree and not in a single group. This supports the CEUU-hypothesis in that it is insufficient to determine the systematic positions of hypotrichs solely by their ventral cirral pattern [43]. Future studies should combine dorsal patterns with molecular analyses to obtain a more robust phylogeny.

## Methods

Saline soil samples (0–10 cm; salinity of soil percolate about 20‰; pH 10.0) were collected in the Longfeng Wetland Nature Reserve (lat. 46°35′30″N, long. 125°13′08″E), Daqing, northern China, on 16 April 2015. Samples were malodorous (very likely due to hydrogen sulphide), and included a large proportion of rotten leaves and branches. For preservation and future isolation, samples were dried at room temperature (about 24 °C) immediately after collection.

Ciliates were stimulated to excyst by applying the non-flooded Petri dish method [22]. They were then isolated and non-clonal cultures were established at room temperature (about 23 °C) in Petri dishes containing filtered soil percolate and squeezed rice grains to enrich the bacterial food.

Living specimens were observed using bright field and differential interference contrast microscopy [31]. Protargol preparation was used to reveal the ciliature and the nuclear apparatus [44]. Counts and measurements of prepared specimens were performed at a magnification of 1,000×. Drawings of protargol-prepared cells were made with the aid of a drawing device (camera lucida). To illustrate the changes occurring during morphogenesis, old (parental) structures were depicted by contour whereas new ones were shaded black. Terminology and systematics basically follow Lynn (2008) [45]; for terms specific for hypotrichs, see references [16, 25, 26, 32]

### DNA extraction, PCR amplification and sequencing

Genomic DNA was extracted from single cells using DNeasy Tissue kit (Qiagen, CA) following the manufacturer’s instructions, with the modification that 25% of the volume suggested for each reagent solution was used. The SSU rRNA gene was amplified according to [46] and [9], using the primers 18S-F (5'-AAC CTG GTT GAT CCT GCC AGT-3') and 18S-R (5'-TGA TCC TTC TGC AGG TTC ACC TAC-3') [47].

### Phylogenetic analyses

A set of 104 SSU rDNA sequences was used in the present study, including the newly obtained sequence of *Heterogastrostyla salina* nov. spec., sequences of 82 related hypotrichs, 19 closely related environmental sequences and two oligotrichs, namely, *Novistrombidium testaceum* and *Strombidium purpureum* as the outgroup taxa (see Fig. 1 for accession numbers). Sequences were aligned in GUIDANCE and ambiguous columns in the alignment were removed with the set parameters (below 0.956), using the GUIDANCE web server [48, 49]. Further modifications were made manually, using BioEdit 7.0 [50]. Ambiguously aligned regions and gaps were excluded prior to the phylogenetic analyses resulting in a matrix of 1,692 characters. Maximum likelihood (ML) analysis was performed, using RAxML-HPC2 v8.2.12, on XSEDE [51] on the online server CIPRES Science Gateway [52] with the GTR + G + I model as the optimal choice. Support for the best ML tree came from 1,000 bootstrap replicates with the GTR + CAT model. Bayesian inference (BI) analysis was performed with MrBayes v3.2.6 on XSEDE [53] on the online server CIPRES Science Gateway, using the GTR + I + G model as selected by MrModeltest v.2.0 [54]. Markov chain Monte Carlo (MCMC) simulations were run with two sets of four chains for 2,000,000 generations with a sampling frequency of 100 and a burn-in of 5,000 trees (25%). All remaining trees were used to calculate posterior probabilities using a 50% majority rule consensus. TreeView v1.6.6 [55] and MEGA 4.0 [56] were used to visualize the tree topologies. For interpretation of bootstrap values we follow Vd’ačný and Rajter (2015); that is, we consider values ≥95 as high, from 70–94 as moderate, from 50–70 as low, and <50 as representing no support [57]. Bayesian posterior probability values <0.95 are considered as low and values ≥0.95 as high [58].

## Data Availability

Sequence data are available in GenBank (Accession Number: MT739409). The datasets used and/or analyzed during the current study are available from the corresponding author on reasonable request. One permanent slide containing the protargol-impregnated holotype specimen of *Heterogastrostyla salina* nov. gen., nov. spec. circled by ink is deposited in the Natural History Museum, London (registration number NHMUK2020.4.4.1). One paratype slide is deposited in the Laboratory of Protozoology, Ocean University of China (OUC, registration number: Leo2015041601).

## Abbreviations

18S rRNA: Small subunit ribosomal RNA
BI: Bayesian inference
bp: Base pairs
DK: Dorsal kinety
FVT: Fronto-ventral-transverse cirri
GC: Guanine-cytosine
GTR + I + G: General time reversible + invariable sites + gamma model of nucleotide substitution
ML: Maximum likelihood
nov. spec.: Nova species
PCR: Polymerase chain reaction
spp.: Species (plural)
